# Characterization of voltage-gated ionic currents in a peripheral sensory neuron in larval *Drosophila*

**DOI:** 10.1186/1756-0500-3-154

**Published:** 2010-06-02

**Authors:** Amit Nair, Michael Bate, Stefan R Pulver

**Affiliations:** 1 Department of Zoology, University of Cambridge, Cambridge CB2 3EJ, UK

## Abstract

**Background:**

The development, morphology and genetics of sensory neurons have been extensively studied in *Drosophila*. Sensory neurons in the body wall of larval *Drosophila *in particular have been the subject of numerous anatomical studies, however, little is known about the intrinsic electrical properties of larval sensory cells.

**Findings:**

We performed whole cell patch recordings from an identified peripheral sensory cell, the dorsal bipolar sensory neuron (dbd) and measured voltage-gated ionic currents in 1^st ^instar larvae. Voltage clamp analysis revealed that dbds have a TEA sensitive, non-inactivating *I*_*K *_type potassium current as well as a 4-AP sensitive, inactivating *I*_*A *_type potassium current. dbds also show a voltage-gated calcium current (*I*_*Ca*_) and a voltage-gated sodium current (*I*_*Na*_).

**Conclusions:**

This work provides a first characterization of voltage-activated ionic currents in an identified body-wall sensory neuron in larval *Drosophila*. Overall, we establish baseline physiology data for future studies aimed at understanding the ionic and genetic basis of sensory neuron function in fruit flies and other model organisms.

## Background

Muscles, central neurons, and peripheral sensory cells all play important roles in coordinating locomotion. To understand how a locomotor system functions, it is crucial to understand the anatomy of each component. But just knowing the anatomy is not enough. Since each component is composed of functionally linked, interdependent excitable cells, it is also essential to understand the underlying electrical properties of cells in all three network components

In *Drosophila*, a number of studies, performed in culture, have identified the different categories of voltage gated ion channels underlying intrinsic properties in neurons [1-5]. However, since these studies were done in culture, the identity of individual neurons could never be determined. Moreover, the characteristics of cultured neurons may not be representative of neurons *in vivo *[4-6]. Several studies addressed this problem in adult flies and developed techniques for recording from identified groups of neurons in acutely dissociated [7,8] and semi-intact preparations [9,10].

A separate line of work has focused on the embryonic and larval development of transmitter responses and intrinsic membrane currents in both muscles [11,12] and motor neurons [6,13,14]. In contrast to adult flies, to date, very little work has been done to characterize intrinsic properties of cells in the larval peripheral nervous system (PNS). The genetics and morphology of these cells have been studied in detail [15-17], yet researchers have lacked even the most basic information about the complement of ionic currents in larval PNS cells.

The dorsal bipolar dendrite sensory neurons (dbds) provide attractive targets for the study of sensory neuron physiology in larval *Drosophila*. Their anatomy has been extensively characterized [17,18]; furthermore, dbd cell bodies are easily identifiable at all developmental stages and accessible to electrophysiological recording. For these reasons, we targeted larval dbds for voltage-clamp analysis. The objective of the present study was to characterize the complement of voltage-activated currents of dbd neurons. Overall, we find that dbd neurons express multiple voltage-activated currents similar to those observed in the central nervous system (CNS) of *Drosophila *larvae.

## Methods

### Fly stocks and animal care

Oregon-R flies were used for all electrophysiology experiments. To image dbd neuron morphology, we used larvae expressing green fluoresecent protein (GFP) in motor neurons and sensory neurons (genotype: C380-GAL4; UAS-mcd8GFP;+;+). Adults were reared at 25°C on standard media. Animals were kept on a roughly 12:12 light dark cycle.

### Larval dissection

We dissected 1^st ^instar larvae in physiological saline on Sylgard (Dow Corning, USA) coated cover slips as published previously [6]. Briefly, we positioned each larva dorsal side up, then used cyanoacrylate glue (Histoacryl, Braun, Melsungen, Germany) to fix the head and tail to the surface. Electrolytically sharpened tungsten needles were used to make an incision along the animal's dorsal surface. Gut and fat bodies were removed with gentle suction from a mouth pipette, then the cuticle was glued flat to the substrate. Care was taken not to disturb the CNS, visible nerves and body wall musculature.

### Whole cell patch electrophysiology

Dissected preparations were mounted on the stage of a BX50WI compound microscope (Olympus, Center Valley, PA) in a custom-made plexiglass recording chamber. dbd neuron cell bodies were identified visually under a 63× water immersion lens. Muscles and neural sheath tissue covering dbd cell bodies were dissolved by local application of 0.2% protease (type XIV, Sigma-Aldrich, Dorset, UK) with a suction pipette as described previously [6]. For maneuvering desheathing pipettes, we used a Narishige MHW-3 hydraulic micro-manipulator (Narishige International, London, UK).

For voltage clamp measurements of total whole-cell current, external solution contained (in mM): 135 NaCl, 5 KCl, 4 MgCl_2_, 2 CaCl_2_, 5 TES, and 36 sucrose, pH 7.1-7.2. CaCl_2 _was omitted and 1 μM TTX was added to the above solution to measure K^+ ^currents. External solution for Ca^2+ ^currents consisted of (in mM) 50 NaCl, 6 KCl, 50 BaCl_2_, 10 MgCl_2_, 10 glucose, 50 TEA-Cl, 10 HEPES, 10 4-AP, and pH 7.1-7.2. For Na^+ ^current measurements, external saline was (in mM): 100 NaCl, 6 KCl, 2 MgCl_2_, 2 CaCl_2_, 0.2 CdCl_2_, 10 sucrose, 50 TEA-Cl, and10 4-AP, pH 7.1-7.2. For measurements of total whole-cell current, internal solution consisted of (in mM): 140 KCH_3_SO_3_, 2 MgCl_2_, 2 EGTA, 5 KCl, 20 HEPES. Internal solution for *I*_*Ca *_and *I*_*Na *_measurements was the same as above, but with 5 mM CsCl_2 _substituted for KCl.

5-10% rhodamine dextran was added to patch pipette tips in initial experiments to confirm the identities of patch-clamped dbds. We also examined dbd morphology using larvae expressing GFP in dbds. Dye filled and GFP labeled dbds were imaged with a AxioCam MRm digital camera (Carl Zeiss Ltd., Hertfordshire, UK) mounted on an Axiophot compound microscope (Carl Zeiss). Images were acquired using AxioVision 4 software (Carl Zeiss).

Whole cell voltage and current clamp recordings were performed with an Axopatch 1D amplifier (Molecular Devices, Sunnyvale, CA). Pipette resistances were 12.5-25 MΩ. Only cells with input resistances > 1GΩ were used for analysis. Mean ± SEM input resistance was 10 ± 1GΩ. For voltage clamp analysis, leak currents were subtracted before current measurements. All traces were sampled at 20 KHz and were digitized, stored and analyzed using pClamp 8.0.2 software running on a Dell desktop PC. Data were plotted using standard features in Excel (Microsoft, Redmond, WA). Final figures were made in Canvas 9 (Deneba, Victoria, CA).

## Results

### Larval dbd neurons generate action potentials and express multiple voltage-gated currents

**Figure 1:**
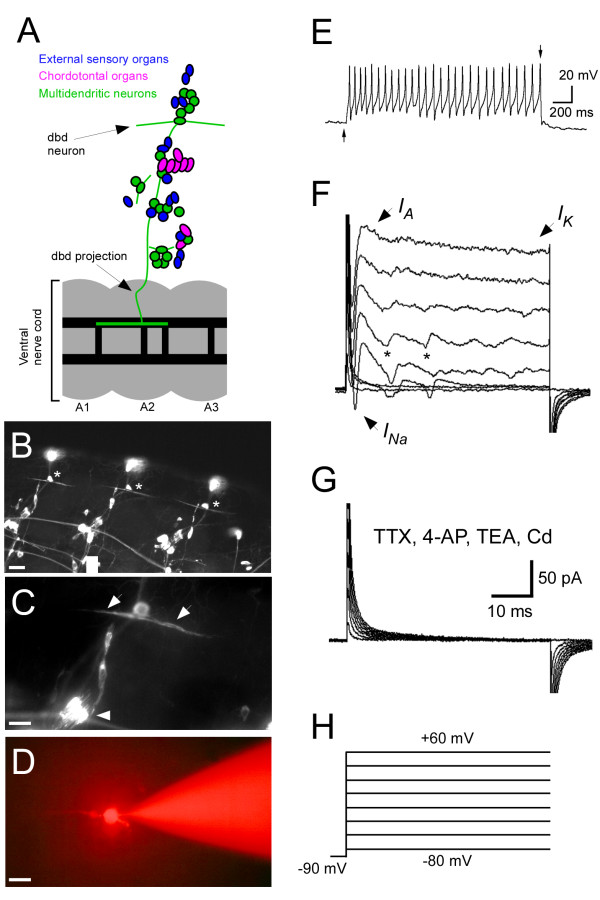
**Larval dbd neurons generate action potentials and express multiple voltage activated currents**. A) Schematic of dbd neuron location in body wall relative to other identified sensory neurons (based on positions detailed in [15]). B) Fluorescently labeled sensory neurons in the body wall of a 1^st ^instar animal. Dorsal is towards top. Asterisks indicate dbd cell bodies in multiple body wall segments. Scale bar = 20 μm. C) High magnification view of a single dbd neuron. Arrows indicate bipolar dentrites. For comparison, chordotonal sensory cells are shown in bottom left (arrowhead). Scale bar = 10 μm. D) Patch pipette filing single 1^st ^instar dbd cell with rhodamine dextran (different animal from B and C). Scale bar = 10 μm. E) Whole-cell current clamp recording from in a 1st instar dbd neuron. The cell generates action potentials in response to a 6 pA current step. Arrows indicate onset and offset of current injection. F) Whole-cell voltage clamp recording from a larval dbd. Features characteristic of inactivating (*I*_*A*_) and non-inactivating (*I*_*K*_) K^+^, channels as well as Na^+ ^channels are visible. Asterisks indicate unclamped action currents. G) In the presence of blockers specific for *I*_*A *_(4-AP, 10 mM), *I*_*K *_(TEA, 50 mM), Ca^2+ ^(Cd, 0.2 mM), and Na^+ ^(TTX, 1 μM) channels, all outward and inward currents are abolished. H) Voltage clamp step protocol.

To provide a baseline for future studies of sensory neuron function and development in *Drosophila*, we have developed a method for performing whole-cell patch clamp recordings from sensory neurons in the body wall of *Drosophila *larvae. As a first step, we have measured ionic currents in one type of larval sensory cell, the dbd neuron. Figure 1A shows a schematic of the location of various types of peripheral sensory neuron in the larval body wall. dbd neurons are shown in green; their dendrites span each hemi-segment in the dorsal muscle field. dbd cells send axonal projections through the intersegmental nerve to the dorsal and ventral neuropil regions of the ventral nerve cord [15,19]. For clarity, the dbd projection path is the only sensory projection path shown in Figure 1A. Figure 1B shows a 1^st ^instar animal expressing GFP in motor neurons and body wall sensory neurons. dbd cell bodies and distinctive bipolar dendrites are visible in multiple body segments (asterisks). Figure 1C shows a high magnification view of the center-most dbd in (B). dbd biopolar dendrites are visible (arrows); dbd morphology is distinct from that of other sensory cells (e.g. chordotonal organs, arrowhead). Figure 1D shows a 1^st ^instar dbd (left) in the process of being filled with rhodamine dextran contained in a patch pipette (right). Because of their distinctive dendritic morphology, individual dbd neurons were readily distinguishable from other cells under DIC illumination and easily targeted for recording.

Figure 1E shows a whole cell current clamp recording from a larval dbd neuron. In response to depolarizing current injection, the cell fires action potentials. Figure 1F shows current traces in response to a series of voltage clamp steps in a larval dbd neuron. Depolarizing steps from -90 mV to 60 mV in 20 mV increments evoke transient outward currents and sustained slow outward currents typical of *I*_*A *_and *I*_*K *_type K^+ ^channels, respectively. These large outward currents dominate the cellular response in these conditions; as a result, slow inward Ca^2+ ^currents are obscured. However, before outward currents predominate, fast inward currents typical of voltage activated Na^+ ^channels are visible (arrow). At some voltage steps, unclamped action currents are visible (Figure 1F, asterisks). The presence of these events suggests that voltage control in dendritic and/or axonal compartments of the neurons is incomplete. As in other neuron types with complex cellular geometries, measurement of voltage-activated currents in the dbd soma may be affected by unclamped currents in distant cellular compartments. All whole cell currents were abolished in the presence of 1 μM TTX (Na^+ ^channel blocker), 10 mM 4-AP (*I*_*A *_blocker), 50 mM TEA (*I*_*k *_blocker), and 0.2 mM external cadmium (Ca^2+ ^channel blocker) (Figure 1G). Figure 1H shows the voltage clamp step protocol used in Figures 1F, G.

### Voltage-gated potassium current

**Figure 2:**
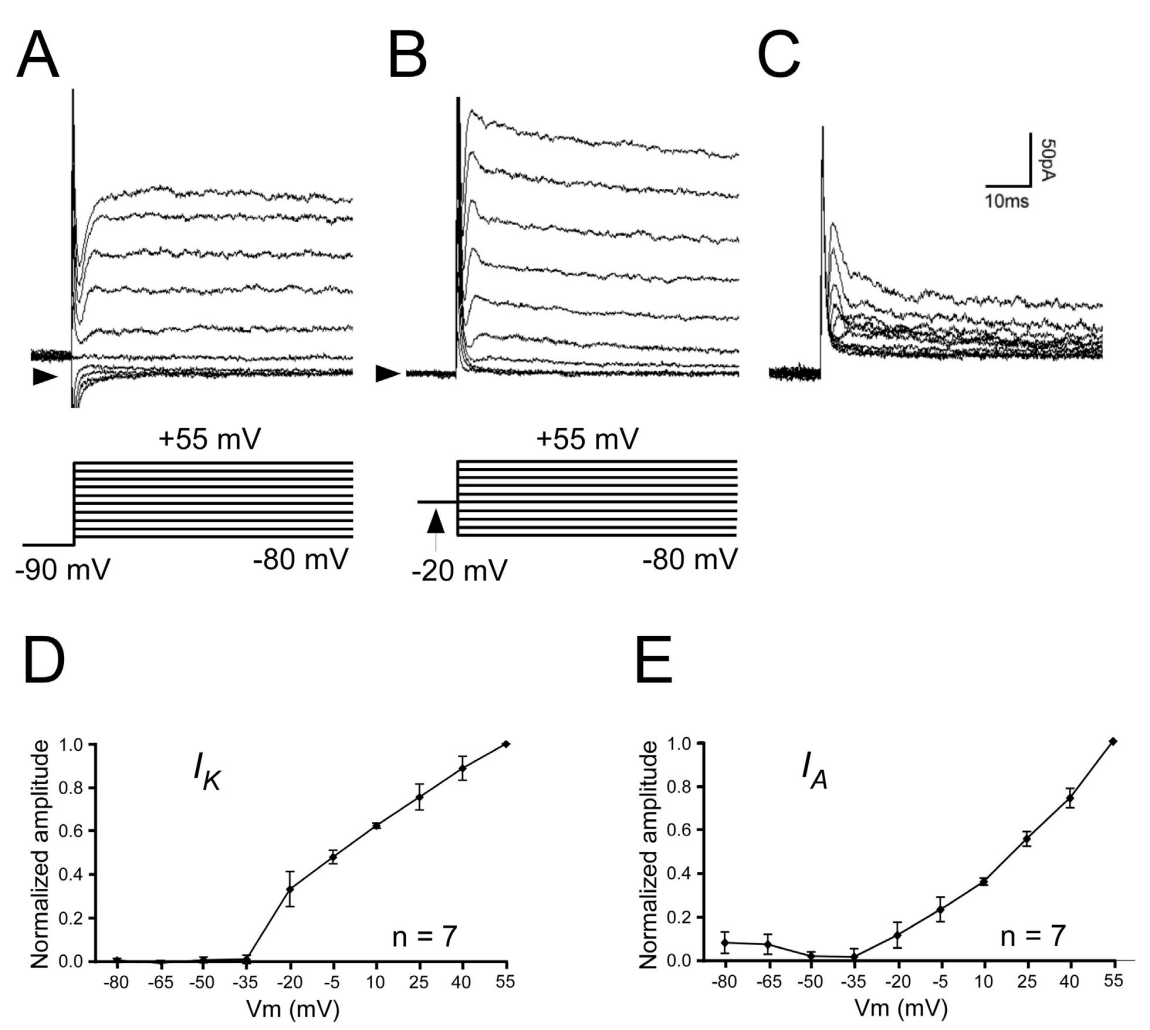
**Both non-inactivating (*I*_*K*_) and inactivating (*I*_*A*_) K^+ ^currents are present in larval dbd neurons**. A) Whole cell recordings from a 1st instar dbd showing *I*_*K *_in response to depolarizing voltage steps (shown below traces). A 500 ms, -20 mV pre-pulse has been used to inactivate *I*_*A*_. B) Response to same voltage steps, but with a -90 mV pre-pulse to release *I*_A _from inactivation. In (A) and (B), arrowheads indicate current level measured at -80 mV. C) Subtraction traces: for each voltage step, subtracting current in (A) from current in (B) yields *I*_*A*_. D, E). Normalized (*I*/*I*_*max*_) I-V relationship of *I*_*K *_(D) and *I*_*A *_(E). Both *I*_*K *_and *I*_*A *_begin activating at -35 to -20 mV. Data plotted as mean ± SEM. External solution for all experiments contains TTX (1 μM) and 0 mM Ca^2+^.

Our initial experiments revealed that dbd neurons display prominent transient and persistent outward currents upon depolarization. To examine the outward K^+ ^currents underlying these responses, we performed recordings in saline containing 1 μM TTX and 0 mM Ca^2+ ^to block Na^+ ^channels and Ca^2+ ^channels, respectively. In order to separate individual K^+ ^currents, we exploited the differential voltage dependence of inactivation between *I*_*k *_and *I*_*A*_. To isolate *I*_*k*_, we held dbd resting potential at -20 mV, then measured evoked currents through a range of voltages from -80 to +55 mV in 15 mV steps. Previous work has shown that *I*_*A *_is inactivated at -20 mV in embryonic and larval motor neurons [6,13]. Figure 2A shows an example of typical *I*_*k *_currents in a larval dbd; the responses show slow activation and little or no signs of inactivation. Previous work has shown that *I*_*A *_in embryonic motor neurons is released from inactivation at -90 [6]. To measure *I*_*A*_, we held dbds at -90 mV, then stepped to the same voltages used to measure *I*_*k *_(Figure 2B). Subtracting *I*_*k *_at each step revealed current due to *I*_*A *_channels. (Figure 2C). To evaluate the fraction of *I*_*k *_inactivation at -20 mV, we compared steady-state current levels at the end of *I*_*A *_subtraction traces to measured *I*_*k *_currents. We found that the fraction of *I*_*k *_inactivation was under 1% in all preparations. Figure 2D, E shows current voltage (I-V) relationships for *I*_*k *_and *I*_*A*_, respectively. Currents are normalized to maximal current (*I*/*I*_*max*_). I-V plots show that both *I*_*k *_and *I*_*A *_begin activating between -35 and -20 mV (n = 7).

### Voltage-gated calcium current

**Figure 3:**
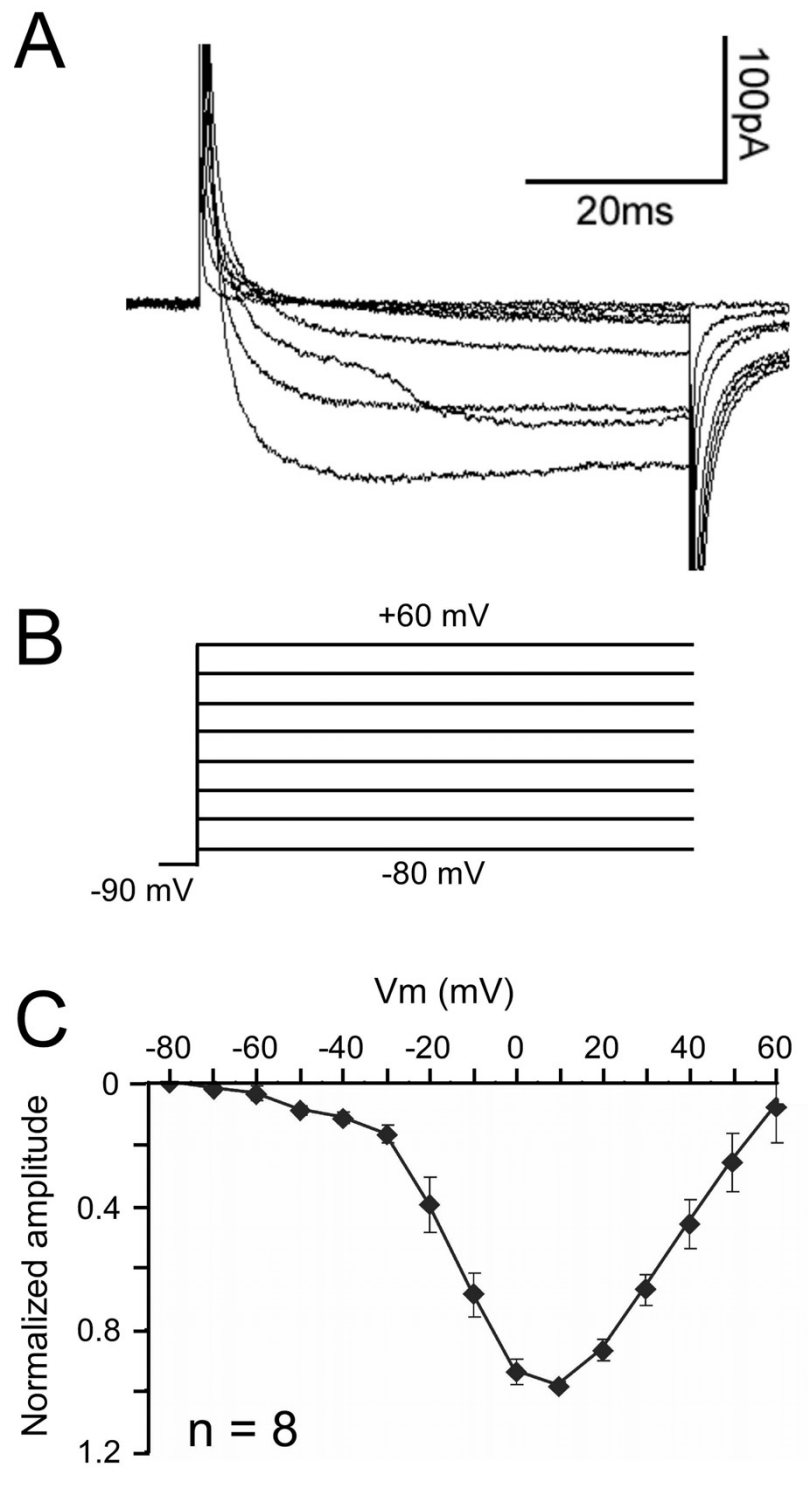
***I*_*Ca*(*Ba*) _expression in a first instar larval dbd neuron**. A) Whole cell voltage clamp recording from dbd neuron. B) Voltage step protocol for traces. Currents are evoked from voltage steps between -80 mV to +60 mV in increments of 10 mV. For clarity, 20 mV increments are shown in (A, B). The voltage step protocol is preceded by a -90 mV pre-pulse to remove any inactivating components. C) Normalised I-V relationship of *I*_*Ca*(*Ba*) _the channels activate between -50 and -40 mV and peak between 5 and 15 mV. Data plotted as mean ± SEM. External solution contains 4-AP (10 mM), TEA (50 mM), and TTX (1 μM). Ba^2+ ^is substituted for Ca^2+^. Internal solution contains cesium (5 mM).

Under normal recording conditions, outward K^+ ^currents overwhelm inward Ca^2+ ^currents during voltage clamp experiments. Therefore, to measure *I*_*Ca *_in dbds, we performed voltage clamp experiments with pharmacological blockers for *I*_*k *_(50 mM TEA) and *I*_*A *_(10 mM 4-AP) and Na^+ ^(1 μM TTX) channels in the external solution. We also substituted cesium for K^+ ^in our internal solution to internally block K^+ ^channels. Finally, we substituted Ba^2+ ^for Ca^2+ ^in our bath solution to prevent activation of Ca^2+ ^dependent K^+ ^channels. Ba^2+ ^also prevents Ca^2+ ^channel inactivation. It is important to note that because of the presence of Ba^2+ ^in our external solution, the currents we measure in these experiments are not Ca^2+ ^currents *per se*; previous work has used *I*_*Ca*(*Ba*) _currents provide estimates of voltage-gated Ca^2+ ^channel activation parameters [6,20]. Figure 3A shows typical *I*_*Ca*(*Ba*) _currents in response to depolarizing steps from -90 to +60 mV (Figure 3B). At some voltages, unclamped inward currents are visible; this is probably caused by incomplete voltage control of distally located Ca^2+ ^channels. Figure 3C shows the I-V relationship for *I*_*Ca*(*Ba*) _in larval dbds. Currents are normalized to maximal current (*I*/*I*_*max*_). The current begins to activate at -50 to -40 mV, and reaches peak amplitude at 5-15 mV (n = 8).

### Voltage-gated sodium current

**Figure 4:**
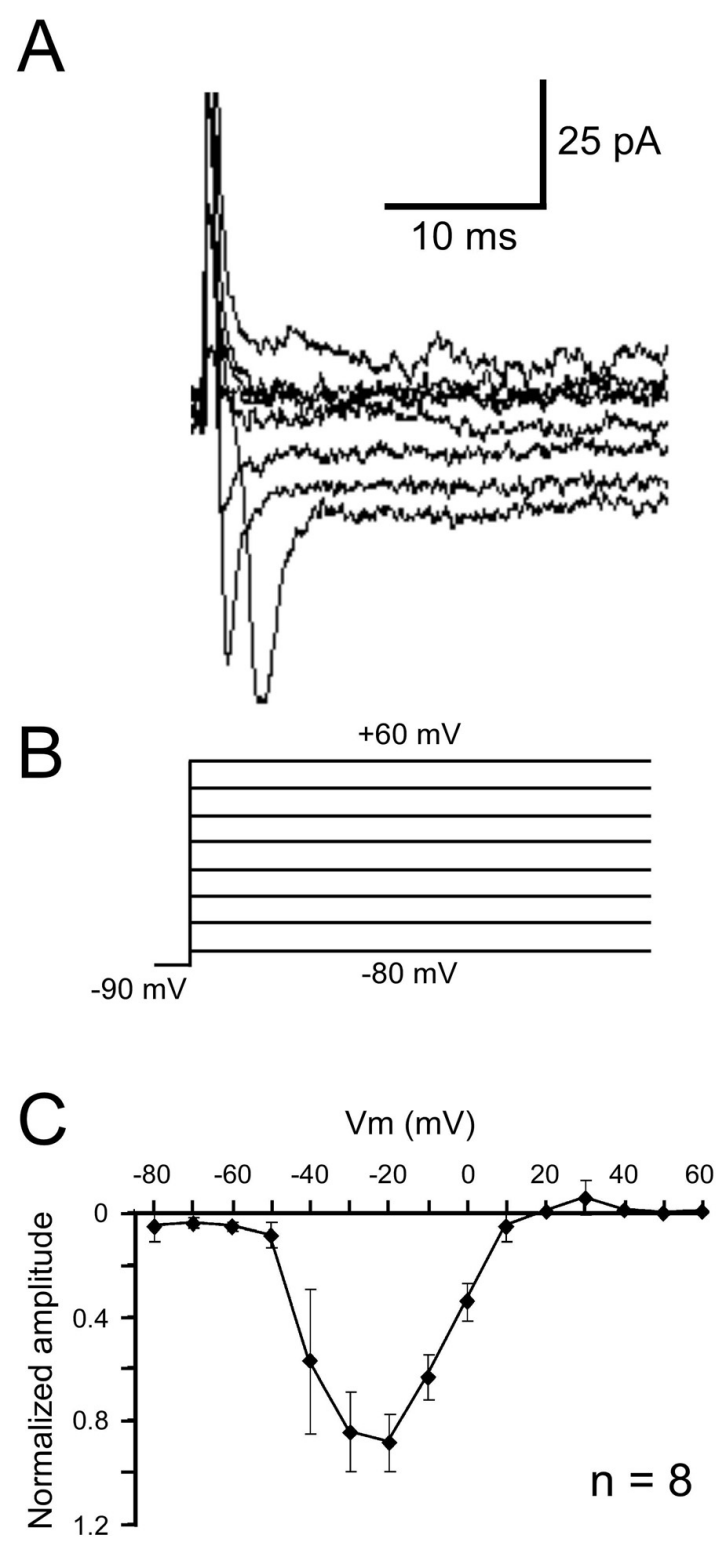
**Larval dbds express voltage gated Na^+ ^channels**. A) Typical Na^+ ^current trace evoked by depolarizing voltage steps. B) Voltage clamp step protocol. Currents are evoked from voltage steps between -80 mV to +60 mV in increments of 10 mV. For clarity, 20 mV increments are shown in (A, B). The voltage step protocol is preceded by a -90 mV pre-pulse to remove any inactivating components. C) Normalized I-V relationship of *I*_*Na*_. The channels begin activating between -50 and -40 mV and peak at -30 to -20 mV. Data plotted as mean ± SEM. External solution contains 4-AP (10 mM), TEA (50 mM) and 0 mM Ca^2+^. Internal solution contains cesium (5 mM).

We isolated *I*_*Na *_by blocking K^+ ^channels with 10 mM 4-AP and 50 mM TEA as well as recording in Ca^2+ ^free saline. Cesium was used instead of K^+ ^in our patch solution to internally block K^+ ^channels.* I*_*Na *_currents sometimes escaped voltage control, indicating that (as in central neurons) spike initiation zones are probably located outside the cell body. However, using online leak subtraction protocols, and careful monitoring of input resistance and voltage clamp parameters, we were able to accurately measure *I*_*Na *_in multiple larval dbd neurons. Figure 4A shows typical Na^+ ^currents evoked by a series of depolarizing voltage steps (Figure 4B). Currents rapidly activated and inactivated within 10 ms. Figure 4C shows the normalized I-V relationship for *I*_*Na *_in larval dbds (n = 8). These data show that in dbds, *I*_*Na *_begins to activate at -50 to -40 mV and reaches peak amplitude at -30 to -20 mV.

## Discussion

In this study, we have presented measurements of voltage-gated ionic currents in dbd, an identified *Drosophila *larval sensory neuron. Larval dbd neurons generate action potentials and express a range of voltage activated channels, including transient and non-inactivating K^+ ^channels, Ca^2+ ^channels, and Na^+ ^channels. Our study represents a technical advance in recording techniques and adds to the growing body of work aimed at understanding the biophysical properties of *Drosophila *neurons.

### Comparison with *Drosophila *motor neurons

dbd neurons and *Drosophila *larval motor neurons both contain a similar complement of voltage gated currents. dbds and motor neurons do not show major differences in activation thresholds for voltage-gated K^+^, Ca^+ ^and Na^+ ^ion channels [6,13,20]. However, detailed quantitative comparison of activation and inactivation parameters in the two cell types is complicated by the fact that neither cell type is electrotonically compact. Distal areas of both cells are difficult to fully control during voltage clamp experiments; this inevitably leads to errors in current parameter measurements. Ionic current parameters aside, dbds do differ from motor neurons in one important respect: unlike motor neurons, dbds do not show any endogenous tonic spiking and/or rhythmic activity (data not shown).

### Function of dbd neurons

The dbds are one of many peripheral sensory neuron subtypes that provide proprioceptive feedback into the larval ventral nerve cord in *Drosophila *during locomotion. This feedback is crucial for generating appropriate locomotor rhythms. Embryos lacking sensory neurons develop, but fail to hatch [16]. When transmitter release is inhibited in the embryonic peripheral (via expression of tetanus toxin), embryos hatch and coordinated locomotor patterns are present, albeit significantly slowed [21]. When feedback from sensory neurons is acutely inhibited in larval life, animals show severe locomotor defects [22]. Conversely, if larval sensory neurons are acutely hyperexcited, locomotion is also inhibited [23]. These and other studies have provided insight into the overall role of PNS neurons, but to date, the function of dbd neurons in *Drosophila *is not clear.

Two lines of evidence suggest that dbds act as stretch receptors in the larval body wall. First, dbd dendrites span the length of each hemi-segment, and are well positioned anatomically to provide information about hemi-segment tension. Second, neurons homologous to dbds are known to be mechanoreceptors in other insects. For example, the stretch receptor organ (SRO) in *Manduca sexta *is composed of segmentally repeating neurons with bipolar dendrites similar to those seen in dbds. SROs fire action potentials in response to mechanical stretching of the caterpillar body wall [24,25]. They appear to provide feedback on the overall tension of each segment during caterpillar locomotion [26]. Whether dbds serve an identical function in *Drosophila *remains an open question.

## Conclusions

Numerous studies have examined how genes influence the development of cellular morphology in the larval peripheral nervous system. But to date, very little work has been done to characterize how these genes affect sensory cell physiology through development. The present study provides a foundation for future work aimed at understanding how gene function regulates both the morphology and cellular physiology of neurons in the peripheral nervous system.

## Competing interests

The authors declare that they have no competing interests.

## Authors' contributions

AN and MB designed experiments. AN performed whole-cell patch experiments and analyzed data. SRP performed anatomy experiments, analyzed data, prepared figures, and wrote the manuscript. All authors read and approved the final manuscript.
